# A Short Review of Advances in MOF Glass Membranes for Gas Adsorption and Separation

**DOI:** 10.3390/membranes14050099

**Published:** 2024-04-25

**Authors:** Zichen Li, Yumei Wang, Jianxin Zhang, Shiqi Cheng, Yue Sun

**Affiliations:** 1 State Key Laboratory of Separation Membrane and Membrane Process, Tianjin Key Laboratory of Green Chemical Technology and Process Engineering, School of Chemistry, Tiangong University, Tianjin 300387, China; 2231030526@tiangong.edu.cn (Z.L.); 2131030496@tiangong.edu.cn (Y.W.); sunyue@tiangong.edu.cn (Y.S.); 2 School of Textile Science and Engineering, Tiangong University, Tianjin 300387, China

**Keywords:** metal–organic frameworks, MOF glass membrane, melt-quenching, gas separation

## Abstract

The phenomenon of melting in metal–organic frameworks (MOFs) has recently garnered attention. Crystalline MOF materials can be transformed into an amorphous glassy state through melt-quenching treatment. The resulting MOF glass structure eliminates grain boundaries and retains short-range order while exhibiting long-range disorder. Based on these properties, it emerges as a promising candidate for high-performance separation membranes. MOF glass membranes exhibit permanent and accessible porosity, allowing for selective adsorption of different gas species. This review summarizes the melting mechanism of MOFs and explores the impact of ligands and metal ions on glassy MOFs. Additionally, it presents an analysis of the diverse classes of MOF glass composites, outlining their structures and properties, which are conducive to gas adsorption and separation. The absence of inter-crystalline defects in the structures, coupled with their distinctive mechanical properties, renders them highly promising for industrial gas separation applications. Furthermore, this review provides a summary of recent research on MOF glass composite membranes for gas adsorption and separation. It also addresses the challenges associated with membrane production and suggests future research directions.

## 1. Introduction

Membrane separation technology plays an important role in the purification and separation of chemicals due to its low separation energy consumption and environmental friendliness. The separation capability can be affected by the chemical structure and subtle physical properties of the membrane material. Metal–organic frameworks (MOFs) are crystalline organic–inorganic hybrid porous polymers assembled based on coordination bonds. MOF compounds exhibit several advantageous characteristics, including porosity with tunable pore dimensions, facile functionalization and the presence of unsaturated metal coordination sites. Based on these properties, MOFs are highly promising for applications across various domains, including catalysis, adsorption and separation processes, molecular recognition, and especially gas separation [1]. However, MOF crystalline materials often contain non-selective defects when prepared as thin membranes, which can significantly reduce their effectiveness in gas separation. MOF glass membranes can effectively alleviate this problem.

**Figure 1:**
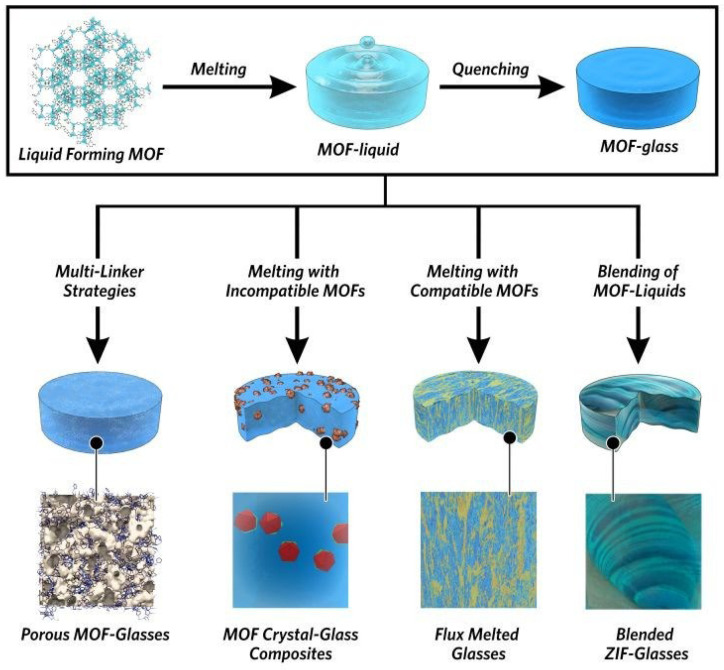
Schematic of the preparation of MOF glass. Reprinted with permission from Ref. [2].

In recent years, there has been a surge in research attention toward the synthesis of MOF glasses through melt-quenching of crystalline MOFs (Figure 1) [2]. During the melting process, the MOF glass loses its long-range ordered structure but retains the short-range ordered units and eliminates grain boundaries. As a result, MOF glass membranes offer the advantage of being easier to prepare and process compared to other MOF membranes. When the melting temperature of MOF is reached, its framework remains stable without undergoing thermal decomposition, allowing the formation of MOF glass upon melting and subsequent quenching. Therefore, the melting temperature (T_m_) of MOFs must be lower than their decomposition temperature (T_d_) [3]. Recently, a variety of materials have been found to be prepared as MOF glass membranes. For example, ZIF-4 has high thermal stability and is structurally similar to SiO_2_ [4]. Bennett et al. were the first to report the thermally induced melting of ZIF-4 under an argon atmosphere [5,6]. Yue et al. successfully prepared the first ZIF-4 glass and proposed the concept of MOF glass [7,8,9,10,11,12,13,14]. Subsequent studies expanded the MOF glass from ZIF-4 to ZIF-62, TIF-4 and GIS systems [6]. These studies have resulted in the creation of various MOF glass systems, including partially fusible ZIF systems, metal-based imidazole phosphate and triazole phosphate systems, and bottom-up synthesized titanium oxide clusters with hydroquinone derivatives [15]. Based on these previous explorations, different fabrication of MOF glass membranes has been developed, including the traditional melt-quenching method, solution phase direct synthesis, ionic liquid-assisted synthesis, ultrasonic and mechanically assisted synthesis, etc. Composite membranes can be fabricated by incorporating various materials using these methods, rendering them highly promising for diverse applications across fields such as gas adsorption and separation [16,17], optics [18,19], catalysis [20], ion conduction [21,22] and sensing [23]. In particular, within the realm of gas separation, MOF glass membranes exhibit significant potential.

Gas separation has relied heavily on low-temperature distillation in traditional industries. However, this process has a high energy consumption and an extended production cycle. In contrast, the emergence of membrane separation technology has garnered attention due to its low energy requirements and high efficiency. A significant milestone in this shift occurred in 1977 when Monsanto successfully applied membrane-based gas separation for hydrogen recovery in petrochemical and ammonia plants [24], marking the advent of a competitive alternative to established separation methods. The diverse pore structures of materials have enabled the production of membranes tailored for various applications. MOF glass membranes offer significant benefits, such as the removal of grain boundaries, long-term stability and high film selectivity. MOF glass membranes can only retain part of their porosity, but this can be optimized via lamination with other materials; hence, they offer significant benefits for gas separation, including the removal of grain boundaries, long-term stability and high film selectivity. The processibility of MOF glass allows it to be re-melted several times after the membranes have been formed, which also makes it easier to repair defects. This characteristic renders MOF glass membranes highly practical for industrial gas separation applications, as they can readily address cracks compared to conventional polycrystalline MOF membranes, which may necessitate more complex defect repair strategies. This review summarizes the recent advances in MOF glass membranes for gas adsorption and separation, highlighting their challenges and future prospects in the field of gas separation technology.

## 2. Crystalline MOF Melting Mechanism

The dominance of crystalline MOFs in research was mainly attributed to their relatively low thermal decomposition temperatures and inability to enter the liquid and glassy states. From a MOF chemistry perspective, it was widely believed that MOF crystals decomposed directly when heated to critical temperatures and could not enter a molten state due to the instability of their organic ligands at high temperatures. A number of MOFs were found to exhibit a molten state, particularly the zeolite imidazolium ester framework structured materials (ZIF), which are an important subset of MOF materials [25]. The discovery of molten MOFs has expanded the processing techniques and chemical applications of MOF materials, opening up the possibility of producing melt-quenched MOF glasses. Amorphous MOFs exhibit several properties, which are absent in crystalline MOFs, including good mechanical properties and processability, offering exciting opportunities for the practical application of MOF materials.

Solid–liquid phase transition is a fundamental and significant phenomenon in materials science. The melt growth process enables the machinability and formability of functional solid-state materials. Melting is the process by which the cohesive forces in a solid relax at a specific temperature. As small molecules, large molecules (polymers), metals and salts melt into liquids, cohesive forces, such as van der Waals forces, hydrogen bonding, metallic bonding and ionic bonding, undergo changes. The melting of solids with covalent bonds as the primary binding force is limited. For instance, crystalline silicon and quartz are examples of covalently extended melting. MOF materials may exhibit multiple cohesive forces simultaneously. This understanding sheds light on the diverse behaviors of materials during phase transitions and their implications for material processing and applications. Despite the increasing importance of MOF materials in materials science and the wide variety of methods available for synthesizing them, their complex structure has resulted in a lack of research into their melting phenomena. The Lindemann melting rule states that the melting of a substance occurs when the amplitude of an atom’s vibrations exceeds the critical distance between the atom and its nearest neighbors, which is approximately 9% [26]. This rule is a widely accepted explanation for the onset of melting in solids. The melting process of organic coordination polymers begins with the rupture of one of the coordination bonds. In MOF systems, the network topology may play a crucial role in determining the melting temperature. This is because topological melting and Lindemann melting may be due to the presence of the same species in different structural forms.

**Figure 2:**
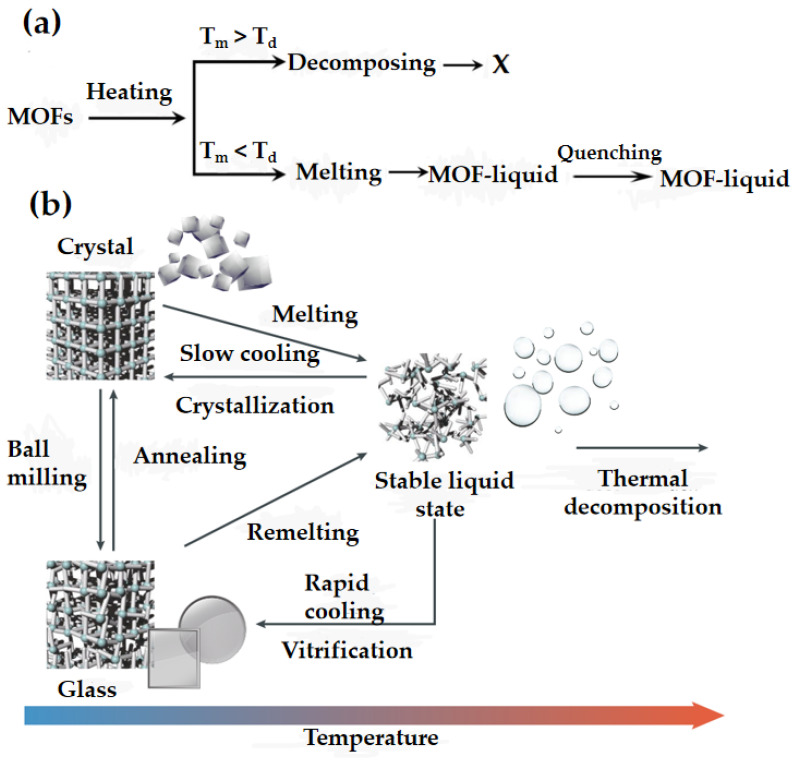
(**a**) Melt conditions of MOF glass. Reprinted with permission from Ref. [3]. (**b**) MOF crystals, liquids and glasses can be fabricated using different approaches. Reprinted with permission from Ref. [27].

The existence of the MOF melting phenomenon relies on a complex equilibrium between the crystal melting temperature and the ligand decomposition temperature (Figure 2a). The ligand’s decomposition temperature is influenced not only by its chemical composition but also by various factors, including the atmosphere, heating rate and particle size. For example, larger particles and faster heating rates in an inert atmosphere may effectively raise the decomposition temperature.

Melt-quenched MOF glasses can be obtained by rapid quenching of MOF crystals from above their melting temperature, resulting in glasses, which are chemically distinct from inorganic, organic and metallic glasses (Figure 2b). The discovery of MOF liquid and glass phases represents a burgeoning field in MOF materials research, which has gained prominence in recent years. By incorporating multiple inorganic or organic ligands in MOF crystals through solvothermal or mechanochemical synthesis processes, multifunctional crystalline materials have been successfully developed. This advancement enables the production of new MOF glasses with tailored chemical functions, akin to alloys, blends and ceramics. MOF glass offers significant advantages over other amorphous materials for various practical applications.

## 3. MOFs for Glass Formation

The majority of MOF crystals undergo framework collapse and irreversible thermal decomposition when heated, without transitioning into a molten state. This phenomenon is usually attributed to the stronger coordination bonds between the metal nodes and the organic linker in comparison to the covalent bonds within the linker. The influence of ligands and metal ions on the formation of MOF glasses is paramount. Various combinations of ligands and metal ions can impact the structure, stability and optical properties of MOF glasses. For example, different ligands can lead to variances in the pore structure and surface chemistry of MOF glasses, while diverse metal ions may affect the conductivity, magnetic properties and other characteristics of MOF glasses. Therefore, the design and synthesis of MOF glasses necessitate meticulous selection and modulation of ligands and metal ions to achieve the desired properties and applications. The commonly employed MOFs for fabricating MOF glass membranes for gas separation encompass zeolitic imidazolate frameworks (ZIF), University of Oslo (UIO), Materials of Institute Lavoisier (MIL) and porous coordination polymer (PCP). Louis et al. reported that metal ions exert a significant impact on the decomposition temperature of ZIF-62 (consisting of Zn^2+^ or Co^2+^ and imidazolate-type linkers), with Zn^2+^ as the metal ion providing higher thermal stability to the material (~510–550 °C for the Co-ZIFs, ~550–600 °C for the Zn-ZIFs). Modulating the imidazole to benzimidazole ratio in ZIF-62 allows for precise control over pore size, thereby optimizing the adsorption and separation of hydrocarbon gases [28]. Four coordination polymers (CPs) were synthesized by Li et al. using various metal ions (Zn^2+^, Cd^2+^, Cu^2+^, Mn^2+^), dmbIm and phosphoric acid (H_3_PO_4_). All four CP glasses had low glass transition temperatures, low viscosities, and Zn^2+^ Cp glass membranes had smaller pores due to stronger binding energies with the ligands, and thus, smaller pores. Glass membranes can be produced at reduced melting temperatures via hot-casting or hot-pressing, exhibiting outstanding screening capabilities for H_2_ and other large-molecule gases [29]. UiO-66 is a MOF comprising Zr^4+^ and substituted aromatic carboxylic acid ligands. This particular MOF is renowned for its meticulously organized amorphous structure comprising metals and ligands, alongside its exceptional chemical stability, which renders it valuable for applications, including gas storage, catalysis and drug delivery. MIL-53 is composed of infinite chains of AlO_4_(OH)_2_ octahedra linked together by 1,4-benzenedicarboxylate groups. With the ability to adjust the pore structure and surface chemistry, it can be utilized for the adsorption and separation of various gases. Both UIO-66 and MIL-53 can be blended and ball-milled with ZIF-62, followed by melt-quenching to create composite glass membranes with adsorption capabilities for CO_2_ [30]. A range of composite glass membranes can be fabricated to cater to diverse separation requirements by utilizing molten MOFs or by blending and fluxing them with other materials. The functionalization of MOF glass can lead to the emergence of unique properties in the resulting MOF glass membrane. Bumstead et al. utilized an amine-functionalized ligand to promote the synthesis of amine-functionalized ZIF-62 [31]. Subsequently, a post-synthetic functionalization process was employed to facilitate the incorporation of additional functional groups. This approach holds promise for tailoring the pore environment of ZIF-62 glasses with diverse functional groups, thus enhancing the membrane’s separation-selective performance.

## 4. Categories of MOF Glass

In recent years, continuous research on MOF glass has resulted in a number of MOF glass materials. According to their composition, they can be broadly divided into two categories: pure MOF glass and composite MOF glass. Pure MOF glass is a single-phase glass prepared by melting and cooling a fusible MOF, while composite MOF glass is a glass material prepared by melting a fusible MOF with another MOF. A composite MOF glass is a glass material prepared by mixing a fusible MOF with another MOF. Depending on the state of the other MOF after melting, it can be further classified into three categories: blend MOF glasses, MOF crystal-glass composites and MOF hybrid glasses [2]. This section summarizes the preparation strategies of these four MOF glasses and the associated research results.

### 4.1. Pure MOF Glasses

**Figure 3:**
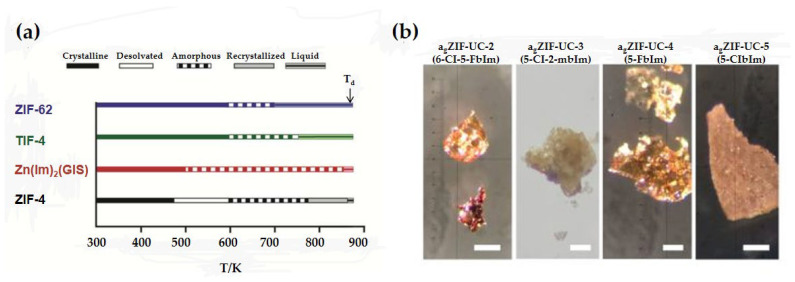
(**a**) Schematic of the thermal events upon heating of four MOF materials. Reprinted with permission from Ref. [6]. (**b**) Microscopic image of the melt-quenched glass samples. The scale bars are 25 µm. Reprinted with permission from Ref. [33].

In recent years, a variety of MOF materials have been reported to exhibit melting and glass transition behavior. The two-dimensional layered MOF material [Zn(1,2,4-triazole)_2_(H_2_PO_4_)_2_)] [32] and the three-dimensional reticulated MOF material ZIF-4 [7] exhibit solid–liquid transitions from crystalline to liquid at 180 °C and 593 °C, respectively, and the corresponding MOF glasses can be fabricated by cooling from the corresponding melting temperatures. In addition to the ZIF-4 mentioned above, which can be prepared via melt-quenching to obtain the corresponding MOF glass, some other MOF materials (Figure 3a) [6], including ZIF-62, Zn(Im)_2_(GIS) and TIF-4, can be melted before decomposition and further converted to MOF glass by quenching, and the melting points of the above MOF materials are 437 °C, 451 °C and 467 °C, respectively. In addition, Hou et al. prepared four new MOFs based on ZIF-62 with similar structures but different chemical environments by using organic ligands modified with different functional groups, and the resulting MOFs exhibited melting properties at different temperatures (Figure 3b) [33]. In particular, the melting temperature of the framework decreased with the increase in electron-withdrawing groups in the framework structure. This result implies that modulation of the melting temperature of MOFs can be achieved via functionalization of organic ligands, which provides a new direction for the development of new MOF glass materials. These pure MOF glass membranes exhibit excellent gas separation properties and possess some mechanical strength.

### 4.2. Blended MOF Glasses

**Figure 4:**
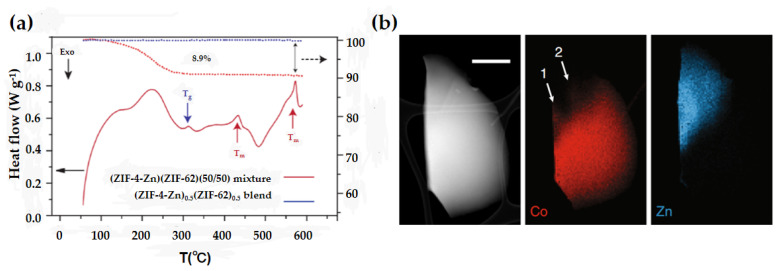
(**a**) DSC curves of the (ZIF-4-Zn)(ZIF-62)(50/50) and the blended glass (a_g_ZIF-4)_0.5_(a_g_ZIF-62)_0.5_. (**b**) Two-dimensional analysis using ADF-STEM showing the particle morphology and EDS chemical maps of Co and Zn. The scale bar is 500 nm. Reprinted with permission from Ref. [34].

MOF blend glasses are prepared by mixing two fusible MOFs, heating to the higher melting point of the two phases and quenching. Both MOF materials—ZIF-4 and ZIF-62—can be transformed into their corresponding MOF glasses via melt-quenching. Based on this, Bennett et al. ball-milled equal amounts of these two skeletal materials and quenched them at 590 °C to obtain the blended glasses of ZIF-4-Co and ZIF-62 (a_g_ZIF-4)_0.5_(a_g_ZIF-62)_0.5_ [34]. The MOF glass exhibits only one glass transition temperature, which indicates sufficient mixing of the two phases; if only the above two glasses were physically mixed, the resulting blend would exhibit two glass transition temperatures (Figure 4a). The structure of MOF glass is characterized by EDX mapping. Relatively sharp interfaces were observed between the Zn and Co domains (Figure 4b). Due to the high viscosity of the two molten phases, only a small area of homogeneous mixing exists. The discovery of blended MOF glasses enables the creation of appropriate pores for separation applications, such as gases, by combining different MOF glass materials.

### 4.3. MOF Crystal-Glass Composites (MOF-CGCs)

**Figure 5:**
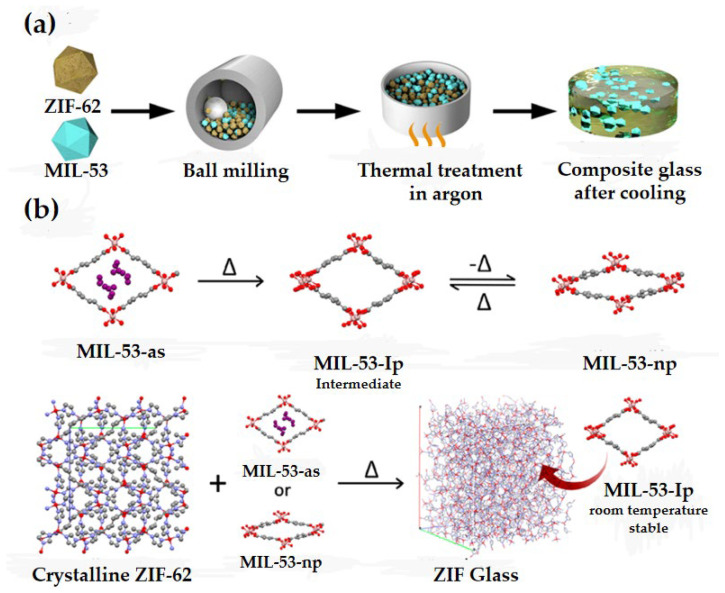
(**a**) Schematic diagram of the CGC fabrication process. Reprinted with permission from Ref. [30]. (**b**) Diagram of the activation process of MIL-53 and the preparation of (MIL-53)_x_(a_g_ZIF-62)_1−x_. Reprinted with permission from Ref. [35].

MOF crystal-glass composites are hybrid glass materials fabricated through the blending and dispersion of a fusible MOF with a non-fusible MOF, followed by melt-quenching. In MOF crystal-glass composites, the crystalline phase of the MOF is dispersed in a matrix of MOF glass, and its structural general formula can be expressed as a_g_[(MOF-1)_x_(MOF-2)_1−x_] (Figure 5a) [30]. The T_m_ of molten MOF is lower than the T_d_ of crystalline MOF. Hou et al. prepared MOF crystalline-glass materials with different MIL-53 loadings by dispersing MIL-53 in ZIF-62 glass: (MIL-53)_x_(a_g_ZIF-62)_1−x_. MIL-53 has good thermal stability and can retain its skeletal structure at the melting temperature of ZIF-62. At the same time, a_g_ZIF-62 acts as a fixative for the backbone of MIL-53, allowing the unstable structure of MIL-53-lp to be maintained at room temperature (Figure 5b) [35]. In addition, when the value of x is greater than or equal to 0.4, the CO_2_ adsorption of the prepared (MIL-53)_x_(a_g_ZIF-62)_1−x_ is higher than the CO_2_ adsorption of any single-component sample. It should be noted that even at low pressures, MOF glass membranes exhibit good CO_2_ adsorption properties. A hybrid matrix membrane with high gas adsorption can be prepared in MOF glass by achieving a relatively high loading capacity of crystalline MOF.

### 4.4. Flux Melted Glass

**Figure 6:**
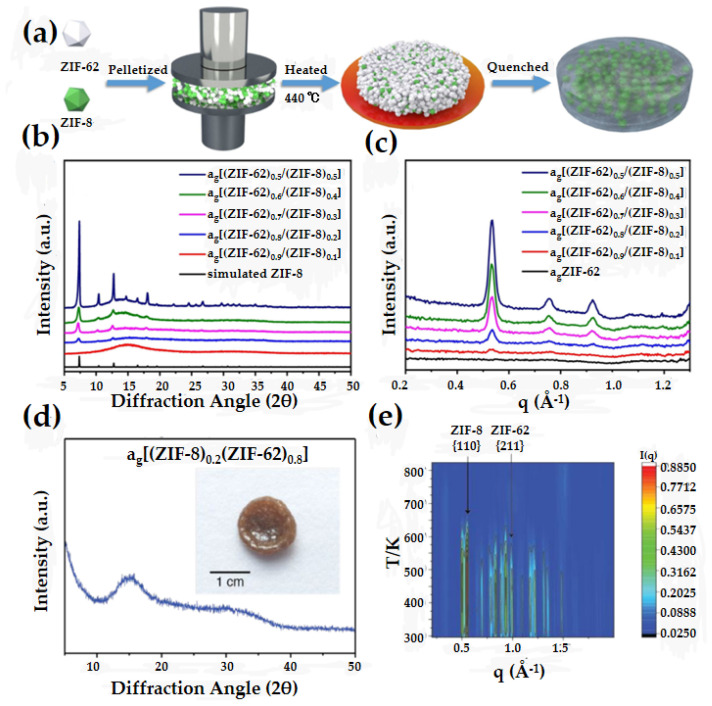
(**a**) The preparation process of flux melted glass membranes. (**b**) XRD of membranes and simulated ZIF-8. (**c**) WAXS of membranes and a_g_ZIF-62. Reprinted with permission from Ref. [36]. (**d**) Powder X-ray diffraction pattern of the glass formed after quenching from 773 K, and (inset) optical image. (**e**) In situ WAXS diffraction of ZIF-8/ZIF-62 flux melted glass. Reprinted with permission from Ref. [37].

Fluxed glasses are prepared in the same way as MOF crystalline-glass composites, with composite glasses prepared by melting a mixture of fusible MOF and a non-directly fusible MOF. Unlike MOF crystalline-glass composites, the XRD of fluxed glass does not show any diffraction peaks of the crystalline material (Figure 6d). In fluxed glasses, both components of the material are converted into glass (similar to blended glasses). ZIF-8 is a MOF material with a decomposition temperature lower than the melting temperature; therefore, it is not possible to prepare the corresponding glass using the direct melt-quenching method. Zhong et al. prepared MOF crystalline-glass materials with different ZIF-8 loadings by dispersing ZIF-8 in ZIF-62 glass. ZIF-8 has good thermal stability and keeps the backbone structure intact at ZIF-62 melting temperature (Figure 6a) [36]. At lower ZIF-8 content, the XRD analysis of the membranes reveals an amorphous nature (Figure 6b). Wide-angle X-ray scattering (WAXS) indicates the absence of the (211) peak of the crystal at ca. 1.0 Å^−1^ in a_g_ZIF-62, implying a transition to an amorphous glassy state (Figure 6c). This membrane has beneficial combined properties, including retention of the porosity of ZIF-62 and the effective mass transfer channels of ZIF-8. Longley et al. prepared an amorphous MOF glass membrane by heating a mixture of ZIF-62 and ZIF-8 to 500 °C in an argon atmosphere, followed by rapid quenching (Figure 6d) [37]. In situ WAXS reveals that the amorphization of ZIF-62 occurs at 600 K, whereas the amorphization of ZIF-8 takes place at 650 K (Figure 6e). Furthermore, the H_2_ porosity of the flux melted glass membranes surpasses that of pure ZIF-62 glass membranes. Essentially, the successful implementation of flux melting offers a pathway to achieving non-melting framework T_m_. Furthermore, the distinctive porosity observed in the flux melted samples indicates potential applications, such as the fabrication of freestanding membranes.

## 5. Structure and Properties of MOF Glass and Composite Membranes

**Figure 7:**
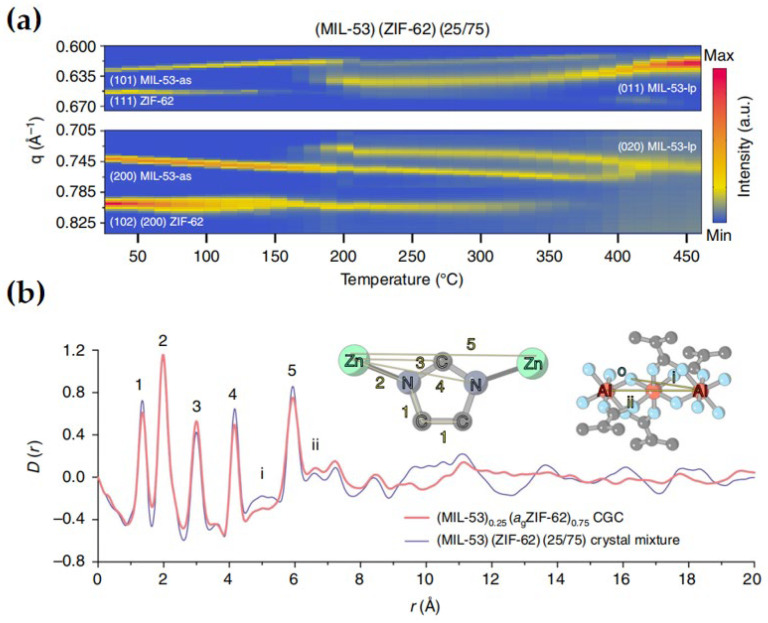
(**a**) Contour plots of in situ synchrotron powder diffraction data were obtained during the thermal treatment process of (MIL-53)(ZIF-62)(25/75) at a heating rate of 10 °C/min^−1^. The Bragg peak hkl indices are indicated for ZIF-62, MIL-53-as and MIL-53-lp. (**b**) The pair distribution function (PDF) D(r), derived from the Fourier transform of the X-ray total scattering structure factor S(Q), was calculated for both the crystal mixtures and CGC. The inset illustrates the PDF peak assignment scheme. Reprinted with permission from Ref. [30].

The presence of inevitable grain boundaries poses a considerable challenge to the synthesis and utilization of MOF films. It is envisaged that MOF glass materials may provide a remedy to overcome these challenges. This facilitates the fabrication of high-performance separation membranes devoid of inter-crystalline defects. The transition from crystalline to amorphous material occurs through the formation of MOF glass upon melting. Powder X-ray diffraction offers insights into the lattice structures of MOFs and their composites. Upon melting of the crystalline MOF, the characteristic Bragg peaks associated with its crystallinity vanish as a result of long-range disorder, leaving behind broad diffusive peaks (Figure 7a). An amorphous MOF glass was characterized utilizing X-ray total scattering and pair distribution functions (PDFs). The short-range ordering and long-range disorder inherent in amorphous materials can be observed and compared with crystalline counterparts (Figure 7b). The absence of intergranular defects in amorphous glass films contributes to their gas separation performance.

The mechanical and structural properties of MOF glasses exhibit unique characteristics, as evidenced by the presence of radial cracks and shear bands in high-Poisson-ratio ZIF-62 glasses. These features distinguish MOF glasses from conventional polymerized structural glasses. The material also demonstrates remarkable micron-scale plasticity, exhibiting lower fracture toughness compared to oxide glass. Moreover, it displays an atypical brittle-tough transition, distinct from that observed in metals and plexiglasses [38,39]. The combination of MOF glass with another glass type, such as inorganic glass, yields a novel amorphous material. The composite exhibits mechanical properties, such as hardness and Young’s modulus, which lie between those of the constituent materials, making it highly suitable for industrial applications [40].

## 6. Gas Adsorption Performance of MOF Glass Membranes

MOF glass membranes introduce novel advancements to gas adsorption applications. Recent findings indicate that melt-cooled MOF glass not only preserves the fundamental metal–ligand bonding characteristic of crystalline MOFs but also retains a significant portion of their porosity. These membranes exhibit exceptional mechanical rigidity and monomer composition, mitigating issues related to compaction observed in powder absorbents. Moreover, they offer simplified regeneration and disposal processes. They also form composites with enhanced gas adsorbing capacity and specific selectivity.

**Figure 8:**
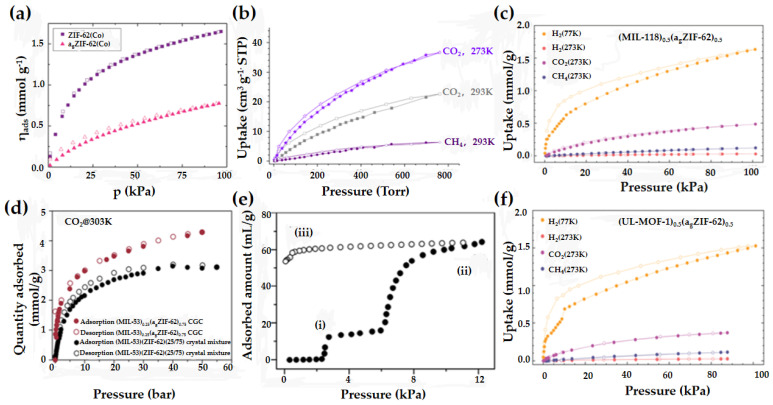
(**a**) CO_2_ gas sorption isotherms of ZIF-62(Co) and a_g_ZIF-62(Co) recorded at 273 K. Reprinted with permission from Ref. [41]. (**b**) Adsorption isotherms of a_g_ZIF-76-mbIm (filled symbols = adsorption, empty symbols = desorption). Reprinted with permission from Ref. [42]. (**c**) Gas adsorption isotherms of samples of (MIL-118)_0.5_(a_g_ZIF-62)_0.5_. Reprinted with permission from Ref. [44]. (**d**) High-pressure CO_2_ adsorption (solid)/desorption (open) isotherms of the crystalline mixture (black) and CGCs (red) performed at 303 K. Reprinted with permission from Ref. [30]. (**e**) Sorption isotherms of benzene vapor on 1-MIG at 25 °C. Solid and open circles represent adsorption and desorption. Reprinted with permission from Ref. [16]. (**f**) Gas adsorption isotherms of samples of (UL-MOF-1)_0.5_(a_g_ZIF-62)_0.5_. Reprinted with permission from Ref. [44].

Louis et al. reported a melt-coolable cobalt-based MOF material (Co-ZIF-62). The a_g_Co-ZIF-62 obtained via melt-quenching not only retains a porosity equivalent to 50% of that of crystalline MOF materials but also shows a very small hysteresis in CO_2_ adsorption, which is very favorable for its application in gas separation or energy storage (Figure 8a) [41]. Zhou et al. studied the permanent pores in a_g_ZIF-76-mbIm [a_g_Zn(Im)_0.93_(5-mbIm)_1.07_]. Compared to the pore volume of its crystalline structure, the pore volume of a_g_ZIF-76-mbIm changes only slightly (from 0.17 vol% to 0.12 vol%), allowing a_g_ZIF-76-mbIm to reversibly adsorb 7 wt% CO_2_ at 273 K and 1 bar (Figure 8b) [42]. This MOF glass membrane maintains high porosity while eliminating intergranular defects, representing a significant improvement for adsorptive gas separation. Henke et al. reported a strategy to regulate the pore size of the corresponding a_g_ZIF-62 by adjusting the ratio of the two ligands in the ZIF-62 framework (Zn(Im)_2−x_(Bim)_x_, x = 0.05, 0.17, 0.35), which was achieved to some extent to control the diffusion rate of propylene gas [28]. The kinetic adsorption results showed a correlation between the benzimidazole content in the framework and the diffusion rate of propylene. This is due to the fact that benzimidazole has a large volume, and as the benzimidazole content in the framework increases, the diffusion rate of propylene within the pore slows down. This means that by adjusting the ligand ratio in these mixed ligand MOF glasses, the diffusion rate of specific gases (e.g., hydrocarbon gases) can be optimized. Separation membranes can be designed to accommodate different gas combinations, as required. Hou et al. have synthesized four isostructural frameworks based on the glassy ZIF-62 structure using the solvothermal method [33]. All ZIF glasses produced through melt-quenching exhibited reversible CO_2_ adsorption, indicating rapid diffusion of guest molecules within their microporous structures. The CO_2_ adsorption capacity of the four MOF glasses is related to their ligands, and the fluorinated ligand enhances its adsorption capacity. By designing various ligands and incorporating specific functional groups, membranes can be created, which are ideal for adsorption separation applications. Lin et al. prepared a bimetallic glass by melt-quenching a cobalt-based zeolite imidazolate framework (ZIF) ZIF-62(Co), which had adsorbed iron coordination complexes [43]. The Fe–N coordination bonds are formed in the MOF liquid. The bimetallic MOF glass demonstrates noteworthy charge transfer efficiency and catalytic activity compared to pure ZIF-62(Co) and a_g_ZIF-62(Co). They improved the CO_2_ adsorption capacity of Co-ZIF-62 glass via modification with Fe ions. This work presents a novel approach to modifying glass films by introducing bimetallic coordination to enhance their properties. A CP/MOF glass, A_g_(mL_1_)(CF_3_SO_3_)], was prepared by Das et al. [16] (Figure 8e). A carbon dioxide gas adsorption analysis of mechanically induced glasses shows their permanent porosity. Moreover, this MOF glass has a good adsorption effect on benzene vapor and will form a glass-to-crystal transition. Bennett et al. prepared two MOF crystal-glass composites (CGCs) by dispersing MIL-53 and UiO-66 in a_g_ZIF-62 [30] (Figure 8d). The interaction between the interfaces of the two phases in the composite membrane makes the open-pore MIL-53 very stable, and it has a higher CO_2_ adsorption than the original material. Two MOF crystal-glass composites were synthesized by adding 50 wt% MIL-118C or 50 wt% UL-MOF-1 to ZIF-62 glass by Ashling et al. [44] (Figure 8c,f). Both CGC membranes exhibited higher H_2_ uptake relative to the pristine material. Furthermore, the CO_2_ isotherms of both CGC membranes exhibited no hysteresis, which was present in the pristine material. These results suggest that MOF CGCs could reach equilibrium more rapidly. In summary, MOF glass membranes exhibit an improved gas adsorption capacity and can be combined with other materials to achieve separations with specific component selectivity.

## 7. Gas Separation Performance of MOF Glass Membranes

**Figure 9:**
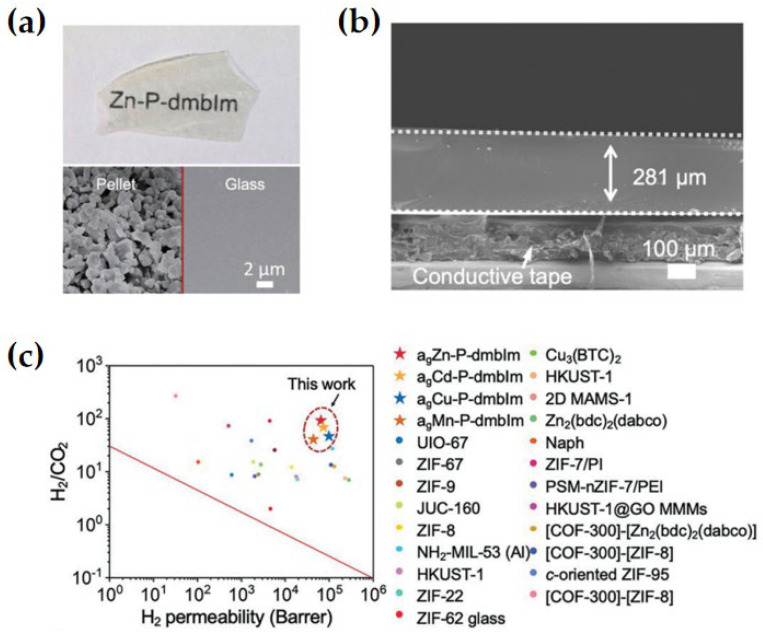
(**a**) Photograph of the Zn-P-dmbIm membrane (top) and scanning electron microscope (SEM) images of the Zn-P-dmbIm pellet and glass membrane (bottom). (**b**) Cross-sectional SEM images of the Zn-P-dmbIm glass membrane. (**c**) Comparison of the H_2_/CO_2_ separation performances of the Zn-P-dmbIm glass membrane with state-of-the-art membranes. Reprinted with permission from Ref. [29].

It is well known that membranes with ultra-high gas permeability and separation selectivity can significantly reduce the cost of industrial gas separation. However, it is difficult to prepare freestanding porous membranes for gas separation applications, especially crystalline MOF materials. In addition, grain boundaries exist between crystalline MOFs, which often increase the gas diffusion resistance and produce non-selective defects. MOF glass formed via melt-quenching of crystalline MOF has become a hot spot in the field of gas separation. During the melting process, MOF glass loses its long-range ordered structure but retains the short-range ordered units and eliminates grain boundaries [45,46]. MOF glass membranes also provide a solution to the non-selective transmission problems of other conventional membranes. Currently, applications for gas separation MOF glass membranes have been reported (Table 1). In 2020, Jiang et al. developed a ZIF-62 glass membrane for selective gas permeation without intergranular gaps using the in situ deposition fusion method, which showed excellent separation efficiency and separation stability for small-molecule gases [47]. The separation factors of the prepared membranes for CO_2_/N_2_, CO_2_/CH_4_ and H_2_/CH_4_ mixtures were 34.5, 36.6 and 50.7, respectively, and they exceeded Robeson’s upper bounds. In 2021, Wang et al. utilized four different coordination polymers to prepare glass membranes following the rapid hot-casting method [29]. The membrane eliminates intergranular defects (Figure 9a). The derived glass membranes have high selectivity for H_2_/CO_2_, H_2_/N_2_ and H_2_/CH_4_, which are well above Robeson’s upper bounds (Figure 9b). Zhong et al. prepared a_g_fZIF-62 using polymer pyrolysis-assisted melting [48]. The gas transport mechanism of methane in the membrane was preferential adsorption/surface diffusion, which leads to high CH_4_ permeability and good CH_4_/N_2_ selectivity. Hou et al. used crystalline ZIF-62 [Zn(Im)_1.95_(bIm)_0.05_] as a filler particle of polyimide for the preparation of hybrid matrix membranes and then converted ZIF-62 to a_g_ZIF-62 via in situ melting [49]. By melting ZIF-62 in situ in a hybrid matrix membrane, ZIF-62 in the liquid form could mend the defects between the MOF and the polymer, resulting in a 23.7% increase in the CO_2_/N_2_ selectivity of the membrane. Mubashir et al. employed non-stoichiometric ZIF-62 MOF glass in conjunction with cellulose acetate (CA) for compounding. Upon doping with 8% ZIF-62 glass, the targeted selectivity of CO_2_/CH_4_ was notably enhanced, exhibiting a substantial increase of 189.3% [50]. Xia et al. fabricated glass membranes utilizing TIF-4, whereby the melting temperature and CO_2_ adsorption capacity were modulated through adjustment of the molar ratio between two ligands, imidazole and 5-methylbenzimidazole. The glass membrane exhibited notable separation factors of 30 for CO_2_/CH_4_ and 27 for CO_2_/N_2_, representing significant enhancements compared to TIF-4 crystals [51]. Zhang et al. employed ZIF-62 to facilitate the melting of ZIF-8 for the synthesis of a mixed glass film, demonstrating exceptional separation performance for C_3_H_6_/C_3_H_8_ [52]. Li et al. introduced ZIF-7 doping into ZIF-62 glass membranes, resulting in enhanced porosity and improved interfacial compatibility. The membranes exhibited notable selectivities for H_2_/CH_4_ and CO_2_/CH_4_, reaching 98.6 and 35.3, respectively [53]. Ma et al. incorporated a fluorine-based linker into a_g_ZIF-62, resulting in the preparation of a_g_ZIF-UC-4 membranes. These membranes exhibited selectivities of 36 and 6.2 for CO_2_/N_2_ and CH_4_/N_2_, respectively. Furthermore, the membranes demonstrated excellent stability under continuous operation and variable pressure test conditions [54]. Feng et al. synthesized a_g_ZIF-62/PIM-1 membranes via an in situ heat treatment process, effectively eliminating non-selective voids between the materials and enhancing the free volume of PIM-1. The membranes exhibited a notable CO_2_/CH_4_ selectivity of 67 [17]. Ao et al. fabricated composite membranes through incorporation of the zeolite material into ZIF-62, resulting in a permeability of 693 GPU for C_4_H_6_. The membranes demonstrated excellent hydrocarbon separation performance [55]. Different from dense and non-porous silicate glass and mesoporous borate glass, MOF glass retains a certain porosity with pore sizes in the sub-nanometer range, which is capable of adsorbing a wide range of gases and demonstrates a certain degree of adsorption selectivity. Therefore, in future development, we can take advantage of the easy molding of MOF glass to prepare a self-supported MOF glass membrane or use it as a continuous phase to prepare a mixed matrix glass membrane. Because of its microporous structure and better processability, it can effectively reduce the difficulty in membrane preparation, which is conducive to the realization of scale-up preparation and industrial applications.

## 8. Summary and Outlook

Recent research advancements have highlighted the effective gas adsorption and separation capabilities of MOF glass membranes, attributed to their favorable properties for gas separation. These properties include easy processing, amorphous characteristics and a unique microporous structure. Such attributes make MOF glass membranes promising candidates for gas separation applications, showcasing their potential for significant contributions to the field of membrane technology. The review discusses the emerging phenomenon of melting in MOFs and its research progress in gas separation membranes. Over the years, various methods of preparing MOF glass membranes have been developed, resulting in membranes with different properties (e.g., blend MOF glasses, MOF crystal-glass composite membranes, MOF hybrid glasses membranes). The functional modification of MOF glass precursors has been shown to effectively enhance membrane separation performance. MOF materials are versatile and flexible, allowing for the modification of properties and functions of MOF glasses through structural modulation. Structural modulation encompasses two key aspects: the control of structural order and the management of porosity, channels and their distribution within the framework. The exceptional characteristics of MOF glass membranes render them highly suitable for the preparation of separation membranes. Consequently, the future research prospects for MOF glass membranes in the field of gas separation appear exceedingly promising.

Despite the notable advancements in recent years, the development of MOF glass membranes remains at an early stage. There are still some challenges in the preparation and application of MOF glass membranes. (1) In order to achieve large-scale preparation and industrial application of MOF glass membranes, further research and optimization of the preparation methods are needed to reduce costs and improve efficiency. The challenge of achieving selective transport properties while synthesizing internal pores with specific dimensions and chemical environments is a major research focus driving the application of glassy MOFs. (2) The preparation of functional glassy MOF materials from crystalline MOFs is a particularly attractive and relatively simple new approach to materials’ fabrication, and although the preparation of designable MOF glasses is still relatively distant, it is expected that the properties and functionality of MOF glasses can be altered via structural modulation of the crystalline material, given the versatility and flexibility of crystalline MOF materials. (3) The stability of MOF glass membranes is one of the keys to its application. Under certain environmental conditions, MOF glass membranes can be structurally damaged or lose their porous nature, affecting the material’s performance and application effectiveness. Therefore, improving the thermal stability and hydrolysis resistance of MOF glass membranes is an important challenge. (4) MOF membrane separation performance is typically evaluated through single-gas permeation tests, which demonstrate ideal gas permeability and selectivity. However, in practical applications, gases exist as mixtures during the production processes, and testing under relevant gas mixture conditions provides more realistic results than single-gas tests. Moreover, industrial gas mixtures often contain various impurities, such as water, hydrogen sulfide and carbon monoxide. Therefore, it is essential to develop stable membranes capable of withstanding complex environmental conditions.

Computational simulation has emerged as a valuable tool for enhancing experimental solutions and has witnessed significant development and widespread adoption in recent years. Computational studies can play a crucial role in predicting membrane stability, assessing the impact of impurities on the multicomponent gas separation performance of membranes and guiding experimental efforts toward the development of suitable membrane candidates.

Melt-quenched MOF glass is a recent discovery in the field of glass science. Further research in this area may provide new insights into unresolved questions and mysteries. As a new type of functional material, MOF glass has broad prospects for industrial application. It is believed that more MOF glasses will be discovered now that the high-temperature thermal behavior of MOF materials has received attention. However, it is important to consider the end goal when exploring the amorphous area of MOFs, as the limitations of the characterization and testing techniques involved in the amorphous area, as well as the diversity and flexibility of MOF materials, make it impractical to attempt to quickly and accurately conduct a comprehensive study to gain an in-depth understanding of this area. The field of amorphous MOFs is expected to introduce new research tools and produce a large number of new functional MOF amorphous materials. MOF glass membranes present notable advantages compared to traditional MOFs, showcasing superior mechanical properties, absence of intergranular defects and enhanced separation efficiency. In the future, MOF glass can play an important role in environmental protection, energy storage, catalytic reaction, etc., with great commercial opportunities.

## 9. Materials and Methods

Some of the sentences in the article were touched up using ChatGPT-3.5 by requesting the touched up request in the AI’s chat box with the sentences we have written, confirming that the generated sentences have the same meaning and that the touched up sentences provide a better reading experience.

## Figures and Tables

**Table 1:** MOF glass membranes for gas separation.

MOF	Method	Permeance (GPU)					Selectivity					Ref.
		H_2_	CO_2_	CH_4_	N_2_	C_2_H_6_	H_2_/CH_4_	CO_2_/N_2_	CO_2_/CH_4_	CH_4_/N_2_	C_2_H_6_/C_2_H_4_	
ZIF-62 glass	In situ	65.67	28.95	1.10	1.22	N/A	50	34	36	N/A	N/A	[47]
Zn-P-dmbim	Hot-casting	6851.52	3805.01	1246.83	1406.74	N/A	5.5	N/A	N/A	N/A	N/A	[29]
a_g_fZIF-62	Melt-quenching	N/A	N/A	30,000	N/A	N/A	N/A	N/A	N/A	6	N/A	[48]
ZIF-62	Hot-casting	N/A	579.82	N/A	26.51	N/A	N/A	21.9	N/A	N/A	N/A	[49]
ZIF-62/ZIF-8	Melt-quenching	N/A	N/A	N/A	N/A	41,569	N/A	N/A	N/A	N/A	7.16	[36]
ZIF-62	Solvent-casting	N/A	84.8	N/A	N/A	N/A	N/A	N/A	35.3	N/A	N/A	[50]
TIF-4	Melt-quenching	N/A	149	N/A	0.549	N/A	N/A	27	30	N/A	N/A	[51]
ZIF-8/ZIF-62	Melt-quenching	N/A	6.5	N/A	N/A	N/A	N/A	34.6	29.9	N/A	N/A	[52]
ZIF-7/ZIF-62	Melt-quenching	120.3	43.1	N/A	N/A	N/A	98.6	N/A	35.3	N/A	N/A	[53]
a_g_ZIF-UC-4	Melt-quenching	N/A	4752	818	N/A	N/A	N/A	36	N/A	6.2	N/A	[54]
a_g_ZIF-62/PIM-1	In situ	N/A	5914	N/A	N/A	N/A	N/A	N/A	67	N/A	N/A	[17]

## Data Availability

Not applicable.
